# Radiotherapy in the Treatment of Subcutaneous Melanoma

**DOI:** 10.3390/cancers13225859

**Published:** 2021-11-22

**Authors:** Valentina Borzillo, Paolo Muto

**Affiliations:** Radiation Oncology Unit, Istituto Nazionale Tumori-IRCCS-Fondazione G. Pascale, 80131 Napoli, Italy; p.muto@istitutotumori.na.it

**Keywords:** subcutaneous melanoma, radiotherapy, hyperthermia, BNCT

## Abstract

**Simple Summary:**

The non-surgical treatment of cutaneous and/or subcutaneous melanoma lesions involves a multitude of local treatments, including radiotherapy. This is often used when other local methods fail, and there are currently no clear guidelines or evidence-based recommendations to support its use in this setting. This review, collecting the retrospective and prospective experiences on radiotherapy alone or in combination with other methods, aims to provide a scenario of the possible advantages and disadvantages related to its use in the treatment of skin/subcutaneous melanoma lesions.

**Abstract:**

Malignant melanoma frequently develops cutaneous and/or subcutaneous metastases during the course of the disease. These may present as non-nodal locoregional metastases (microsatellite, satellite, or in-transit) included in stage III or as distant metastases in stage IV. Their presentation is heterogeneous and associated with significant morbidity resulting from both disease-related functional damage and treatment side effects. The standard treatment is surgical excision, whereas local therapies or systemic therapies have a role when surgery is not indicated. Radiotherapy can be used in the local management of ITM, subcutaneous relapses, or distant metastases to provide symptom relief and prolong regional disease control. To increase the local response without increasing toxicity, the addition of hyperthermia and intralesional therapies to radiotherapy appear to be very promising. Boron neutron capture therapy, based on nuclear neutron capture and boron isotope fission reaction, could be an alternative to standard treatments, but its use in clinical practice is still limited. The potential benefit of combining radiotherapy with targeted therapies and immunotherapy has yet to be explored in this lesion setting. This review explores the role of radiotherapy in the treatment of cutaneous and subcutaneous lesions, its impact on outcomes, and its association with other treatment modalities.

## 1. Introduction

Malignant melanoma is the most common tumor that develops cutaneous and/or subcutaneous involvement during the course of the disease [1].

According to the eighth-edition AJCC melanoma staging system, cutaneous and/or subcutaneous metastases are categorized as non-nodal locoregional metastases (microsatellite, satellite, or in-transit) accounted for in the N category (N “c”) included in stage III or as distant non-visceral metastases (M1a) in stage IV. A microsatellite is defined as microscopic cutaneous and/or subcutaneous metastases that is not clinically identifiable and is found adjacent or deep in a primary lesion. Clinically evident cutaneous and/or subcutaneous metastases are defined as satellite metastases (SMs) when occurring within 2 cm of the primary melanoma and in-transit metastases (ITMs) when more than 2 cm from the primary lesion in the region between the primary melanoma and the regional lymph node basin [2].

In patients with melanoma, the incidence of cutaneous metastases ranges from 2% to 20% and of ITMs ranges from 3.6 to 7.0% [1,3]. 

The typical clinical presentation is a pigmented papule and/or nodule, which can often progress in size and number, ulcerate, become infected, and bleed, leading to significant morbidity and worsening of the patient’s quality of life [1].

Non-nodal locoregional metastases develop as a result of tumor cell invasion through the lymph vessels between the primary tumor site and the drainage basin of the regional lymph nodes. They occur more frequently in the lower limbs, due to the greater lymphatic stasis due to gravity or following a lymphadenectomy, and a longer lymphatic network that leads the cancer cells to accumulate more. Conversely, distant cutaneous metastasis can spread both through multiple lymphatic basins, as occurs in primary trunk melanoma, and through blood vessels [1,4].

Survival is similar between microsatellites, SMs, and ITMs, but may change based on their association with different nodal involvement (five-year survival rates are 83%, 69%, and 32% for stage IIIB, IIIC, and IIID, respectively) [2].

Distant cutaneous or subcutaneous metastases are a distinct entity from non-nodal locoregional metastases, with a poor prognosis (five-year survival <30%) but better than distant metastases from other primary tumors [5].

The different prognoses of the two types of cutaneous/subcutaneous metastases and their heterogeneous presentation (number, site, size, and symptoms) should be taken into account in their clinical management and in the choice of therapeutic options. 

Surgical excision is generally considered first-line therapy when possible. Local therapies (including electrochemotherapy (ECT), intralesional injection, isolated limb infusion (ILI) or perfusion (ILP), carbon dioxide laser ablation, cryotherapy, radiotherapy (RT), and topical therapy) or systemic immunotherapies and/or targeted therapies play a role when surgery is not appropriate [4,6,7,8] 

### 1.1. Radiotherapy Overview

Radiotherapy can be used in local management of ITM, subcutaneous recurrences, or distant metastases when other local first-line treatments have failed or are not indicated. The RT may provide symptom relief and prolonged regional disease control [7]. The choice of dose and fractionation is based on the site, volume, and extent of the lesions, the patient’s performance status, disease stage, and melanoma radiosensitivity [8,9].

The consideration that melanoma is radioresistant rose from the result of initial in vitro studies that demonstrated a high cellular repair capacity at low doses, due to many intrinsic factors, but also a greater sensitivity and therefore a better response to higher doses per fraction [10,11,12].

In the 1980s, clinical radiobiological and observational studies of radiotherapy in the treatment of recurrent and metastatic melanoma lesions (cutaneous included) with different schedules corroborated the laboratory findings [13,14]. These studies showed a significant relationship between dose per fraction and tumor response (59% complete response (CR) for dose > 4 Gy versus 33% CR for dose < 4 Gy), and between tumor volume and the response to RT (86% CR for cutaneous lesions ≤ 1 cm in diameter versus CR < 30% for lesions > 5 cm in diameter or larger) [15,16]. However, no difference in the response rates between the number of fractions (frs) or the treatment time emerged in these studies (65% CR with 9 Gy × 3 frs in 8 days versus 72% CR with 5 Gy × 8 frs in 26 days) [15,16]. The findings in these studies supporting a higher dose per fraction to treat melanoma was questioned by the Phase III Radiation Therapy Oncology Group (RTOG) 83-05 trial. This failed to demonstrate an advantage with large fractions in metastatic melanoma involving any site other than the brain or abdomen (20 frs × 2.5 Gy daily 5 days/week similar to 4 frs × 8Gy at weekly intervals) [17].

The reasons for the disparity between the results of these studies could be the wide range of doses and fractionation used, some of which were probably not optimal for achieving tumor control; the early death of many patients from metastatic disease, which did not allow the effective local response of the tumor to be evaluated; or the different dose per fraction response based on the type of tissue irradiated (e.g., lymph nodes or parenchyma). However, these studies supported the beneficial role of RT in the treatment of melanoma lesions, especially with moderately high-dose and hypofractionated regimens [13,14,15,16,17]. 

It has now been proven that the radiosensitivity of melanoma could be improved with high doses, a short total treatment time, radiosensitizers, tumor reoxygenation induced by fractioned radiotherapy, and high linear energy transfer (LET) [18,19].

Furthermore, thanks to technical advances in planning (CT simulation) and delivery (intensity modulated RT, volumetric modulated arc therapy, stereotactic radiosurgery, hadron therapy), radiotherapy is increasingly able to accurately deliver high doses to the target while sparing surrounding normal tissue, thus obtaining good local control and low toxicity [7,20,21].

Imaging plays a central role in radiotherapy, from diagnosis to treatment planning and monitoring. In recent years, thanks to the development of artificial intelligence software and the use of big data, a very large number of features can be extracted from the images and correlated with clinical results, such as the efficacy or toxicity of the treatment [22]. This is what radiomics does: Using all this information from related images with other relevant data (such as medical notes, pathology, biology, or genomics), it can create predictive models and potentially be used to personalized radiation treatments. Radiomics is rapidly evolving in the radiotherapy field and could have a big impact on daily practice, from cancer diagnosis and staging, treatment simulation, treatment planning and delivery, quality assurance, and predictions of outcomes and toxicity, through to follow-up after treatment [22].

Most of the radiomics experiences in radiotherapy concern the treatment of lung and head and neck cancer, whereas there are none in the treatment of melanoma [23].

Radiomics is not yet used in daily clinical practice because the quality of the published studies is heterogeneous, and there are still many critical issues to be addressed to better define the methods used, the quality criteria, and the way to validate and therefore monitor the results of a predictive model [24].

In the treatment of cutaneous/subcutaneous lesions, radiotherapy has been used in combination with hyperthermia (HT) and intralesional therapies (radiosensitizers or cytostatic agents) to increase its efficacy. 

### 1.2. Radiotherapy and Hyperthermia 

Many uncontrolled studies of combined hyperthermia and RT have suggested a significant advantage of the combination over radiation alone (local response rates between 50 and 80%) [25,26,27,28,29,30,31] (Table 1). The biological rationale for combining hyperthermia with radiation is based on two different mechanisms: hyperthermic radio-sensitization and direct hyperthermic cytotoxicity against radio-resistant cells [27]. The hyperthermia, indeed, may cause an increased blood flow, which may result in an improvement in tissue oxygenation, which then results in increased radiosensitivity. However, the most important mechanism for the interaction of two modalities is the interference of HT with the cellular repair of radiation-induced DNA damage [32,33]. The maximum thermal enhancement ratio for radiation-induced cell kill is obtained when radiation and hyperthermia are applied simultaneously, and can be enhanced by a factor of between 1.2 and 5 [33]. 

Generally, the most important prognostic factors for complete response to combined treatment are radiotherapy dose, tumor size, and minimal thermal parameters (t43 min, Tmin, or temperature exceeded by 90% of thermal sensors). The total number of hyperthermia fractions or the number of fractions per week appears to be irrelevant [34].

The European Society for Hyperthermic Oncology (ESHO) conducted a multicenter prospective phase III randomized trial of RT (3 frs of 8 Gy or 9 Gy in 8 days) with or without adjuvant hyperthermia (43 °C for 60 min) in 70 patients with 134 metastatic or recurrent melanoma lesions [27]. The study demonstrated significantly improved local control (LC) by combination modalities (two-year LC rates: 46% RT + HT vs. 28% RT alone; CR rate 62% RT + HT vs. 35% RT alone) and a clinically derived therapeutic thermal enhancement ratio of approximately 2.0. Moreover, the higher response rate with a dose of 27 Gy rather than 24 Gy and for small tumors (≤4 cm) rather than larger tumors (>4 cm) confirmed that total radiation dose and tumor volume were important prognostic factors. The addition of heat did not significantly increase acute or late radiation reactions. However, because of difficulties with equipment, only 14% of the patients received the prescribed protocol treatment and survival did not differ between the two groups. The five-year overall survival rate was 19% for all patients in contrast to 38% for patients for whom all known diseases were controlled. This underlines the importance of local control in patients with a limited number of metastatic/recurrent melanoma lesions that should be considered for curative therapy.

The correlation between tumor volume, RT dose, and response to treatment has also emerged in other experiences. 

A study on 126 patients with superficial tumors, including 33 with malignant melanoma (MM), showed that the tumor response to RT + HT (mean external beam RT (EBRT) dose of 45 Gy with electrons or photons from 6 or 25 MV and HT to a tumor temperature of 45 °C for 60 min) was better not only for superficial lesions than deep lesions (CR 70% ≤ 3 cm vs. 18% >3 cm; *p* < 0.001), but also for a dose fraction of 3–4 Gy compared to < 3 Gy (CR 77% vs. 55%; *p* = 0.05) [29]. 

For superficial deposits of MM, the CR rate was 36%, and was positively influenced by the size of the dose per fraction > 3 Gy but not by the tumor depth. With an RT dose range of 30–60 Gy, complete responding MM exhibited a mean time to recurrence of 15.0 ± 1.4 months. The response to thermoradiation therapy was dependent on the size of the dose per fraction and on the depth of the lesion. Tumor depth affected not only local control but also survival (16.1 ± 1.2 months for lesions ≤ 3 cm depth vs. 8.7 ± 1.1 months for lesions > 3 cm deep; *p* < 0.001), and the difference in response rate based on the depth of the lesion could not be attributed to any of the thermal parameters. In 126 fields treated with RT + HT, the skin reaction was as follows: none in 42 (33%), erythema in 59 (47%), and transient thermal blisters in 25 (20%). Ulceration occurred in 27 treatment fields but may have been due to direct invasion of the skin by the tumor at the start of treatment. The authors concluded that there was no apparent correlation between maximum tumor temperature, MaxTskin, and skin reactions [29].

Radiation dose per fraction and tumor volume was demonstrated to be critically important in tumor control also in Kim et al.’s experience [28] with 38 patients with multiple recurrent malignant melanoma (subcutaneous lesions and regional lymph node metastases). More than 100 lesions were treated with RT alone and HT + RT, either twice a week or once a week, most with electron beams. The sequence of the combination therapy was to heat the lesions for half an hour at 42.0–43.5 °C immediately prior to RT. The results showed a higher overall tumor response rate following the combination of HT and RT than that obtained with RT alone (70% vs. 46%; *p* ≤ 0.01). However, in an analysis of tumor response rate as a function of dose per fraction and tumor volume, hyperthermia did not give any advantage in the treatment of smaller tumors (less than 10 cm^3^) treated with a high RT dose fraction (≥5 Gy), whereas in larger tumors (greater than 10 cm^3^) it increased the tumor response and regression rate with both large and small RT dose fractions. 

The authors therefore concluded that large melanomas responded better to combination therapy probably due to the selective heating of the tumor and the favorable conditions of the microenvironment of large tumors (chronic hypoxia, reduced pH, and poor nutritional status). After the combined treatment there were no disproportionate skin or subcutaneous reactions, unlike with RT alone, probably thanks to the differential temperatures induced by radiofrequency heating between normal tissues and tumors.

A non-randomized study of about 18 patients with recurrent primary or metastatic malignant melanoma reported the results of the combined RT and HT treatment vs. RT alone of 49 lesions, of which 28 were cutaneous or subcutaneous metastases. The analysis on tumor response as a function of radiation dose per fraction revealed no statistical differences in CR between doses > 4 Gy or ≤ 4 Gy, probably due to a too-small number of patients. However, the addition of hyperthermia to RT positively influenced complete responses at a lower dose per fraction. In the analysis on tumor control as a function of the average tumor dimension, the addition of hyperthermia to RT increased CR more in lesions greater than 3 cm (68.7% vs. 18.5%; *p* = < 0.01) than in those less than 3 cm (51.4% vs. 27.5%; *p* = 0.0026). The study therefore concluded that HT added to RT resulted in higher response rates for larger tumors and that when combined with low-dose-per-fraction RT, it achieved a complete response similar to high-dose-per-fraction treatments [30].

Another old and small study analyzed 24 patients with 48 metastatic malignant melanoma localizations (30 skin, eight subcutaneous, seven nodal, and three other sites) treated with RT + HT (38), RT alone (8), and HT alone (3). Hyperthermia was administered within 30 min following irradiation and RT schedules consisted of either one large fraction (6–8 Gy) once a week in 14–21 days or two fractions (4–5 Gy) twice a week in 21 days with 5 MeV or 250 kV photons or electrons of different energies based on the tumor size. The overall CR rate was 50% in the HT + RT group and 38% in RT alone. The lesions treated with HT alone did not show any response to treatment. The CR appeared to occur more frequently in small lesions, and in the group of patients treated with HT and RT 8 Gy per fraction the CR rate was 83%. The authors, supporting the possible value of combined RT and HT treatment of MM lesions, concluded that tumor response appeared to be related to dose per fraction and metastasis diameter, but due to the study’s numerous biases they could not provide reliable data. [26]

The correlation between tumor volume and response to treatment also emerged in the Radiation Therapy Oncology Group (RTOG) phase III study comparing RT + HT with RT alone in superficial measurable tumors (breast, trunk and extremities, head and neck). The study demonstrated a better response rate with the addition of HT only for lesions < 3 cm in diameter in the breast and trunk/extremities (CR 62% and 67%) without enhancement of acute or late toxicities, probably due to the greater ease of heating smaller lesions. Unfortunately, the number of patients with malignant melanoma lesions included in the study was too small to warrant subgroup analysis [31]. 

### 1.3. Radiotherapy and Intralesional (IL) Therapies 

In a phase II open-label study, 15 patients with clustered in-transit or recurrent dermal and subcutaneous melanoma metastases (98 total target lesions) received intralesional (IL) PV-10 (Rose Bengal 4,5,6,7-tetrachloro-2,4,5,7-tetraiodofluorescein disodium) followed by hypofractionated radiotherapy (30 Gy in 6 frs at 2 frs/week over 3 weeks; photons or electron beams) after previous loco-regional therapy [9]. The radiobiological rationales that may explain the interaction of the two modalities are radiosensitization by PV-10, additive cell kill or augmentation of the host local, and systemic antitumor immune response (including abscopal effect) by regional radiotherapy. The overall response rate (ORR) of patients was 86.6% (CR 33.3% + PR 53.3%) and the clinical benefit was 93.3% (CR 33.3% + PR 53.3% + stable disease 6.7%) in an intention-to-treat analysis. These results also appeared favorable in comparison with those of other experiences with IL therapy alone: IL PV-10 alone (ORR 51%; CR 26%), IL Bacille Calmette–Guérin (BCG) immunotherapy alone (ORR 45%), and talimogene laherparepvec (T-VEC) alone (ORR 26.4%). The 12-, 24-, and 36-month melanoma-specific survival rates from the time of treatment were 77%, 54%, and 40%, respectively. The toxicity, assessed according to the CTCAE V3.0. scale, was grade 1 or 2 in all patients: pain at the injection site (87%), localized edema (73%), injection site swelling (60%), blistering (20%), and erythema (20%). There was only one grade 3 (pain at the injection site) and no grade 4, no treatment interruption, no treatment-related phototoxicity, and no permanent lymphedema. The low toxicity rate and a CR of 74.6% for target lesions ≤ 10 mm support the combination of IL PV-10 and RT as safe and useful especially in patients with multiple low-volume lesions included in an RT field.

Another small study examined the effect of intralesional beta-interferon (IFN-β) injections (3–5 million units per injection administered three times weekly) combined with radiotherapy (40–50 Gy at 1.8 Gy fraction) in 20 patients with metastatic inoperable malignant melanoma. After combined therapy, five patients showed partial remission (30%) and 12 patients complete remission (70%), with permanent regression of metastases. Notably, several of these patients had relatively bulky disease, and the combined treatment saved them from a potentially mutilating surgery and reduced pain [35]. 

An old study investigated the combination of IL BCG injections into metastatic melanoma skin nodules with percutaneous RT in 19 patients. The combination approach appeared to give some advantages over treatment with BCG alone, mostly in patients with numerous small metastases and those with a small number of larger metastases. However, the results of this study should be validated by other studies, because the timing and dosage of both BCG and RT was not well defined and the number of patients was limited [36]. Studies of radiotherapy in combination with intralesional therapies are show in Table 2.

### 1.4. Boron Neutron Capture Therapy (BNCT)

BNCT is a promising cancer treatment based on nuclear neutron capture and boron isotope (10B) fission reaction. When intracellular 10B is irradiated with low-energy thermal neutrons it can produce high LET α particles and lithium-7 isotope nuclei, which are lethal to these cells. Since α particles have very short paths of 5–9 μm (almost the size of a cell), their destructive effects are limited to boron-containing cells. Consequently, the neoplastic cells can be selectively destroyed without damaging the normal cells if they absorb more 10B than normal ones [37]. Theoretically, in this way, BNCT could reduce radiation-related damage to normal tissue, improve patients’ quality of life, and be an alternative to standard treatments. Unfortunately, there is no selective boron delivery agent for BNCT; the only approved boron drugs are boronophenylalanine (BPA) and sodium borocaptate (BSH). On the other hand, for many years the source of neutrons has been specially designed nuclear reactors that have limited the use of BNCT in clinical practice. Recently, the fabrication of accelerator-based neutron sources mounted in the hospital has allowed phase I/II clinical trials to be undertaken [37,38]. 

Although studies on BNCT have begun around the world, the number of melanoma patients treated with BNCT remains limited. 

A phase I/II clinical trial was conducted in Argentina on seven patients with multiple subcutaneous melanoma skin metastases on extremities that received an intravenous infusion of BPA ~14 gr/m^2^ followed by the exposition of the area to a mixed thermal–epithermal neutron beam at a RA-6 reactor (maximum prescribed dose range of 16.5–24 Gy-Eq). Overall response was observed in 69.3% of the nodule valuables, with 30.7% of additional no-changed tumors. No progression of disease was observed inside any treated areas. The toxicity was acceptable, with ulceration in only three out of 10 evaluable areas (30% acute toxicity grade 3), and was found to be related to skin dose and the anatomic area [39].

Another Japanese BNCT experience, with BPA and an epithermal neutron beam, reported the results of eight patients with primary melanoma lesions (cT1-2N0M0) located on the sole or face, who did not undergo surgery. The overall control rate (CR + partial response (PR) without recurrence) was 88% (7/8 patients). Normal skin toxicity, scored according to the Common Terminology Criteria for Adverse Events (CTCAE) v.4.0., was edema, pigmentation, and erosion requiring no painkillers. No adverse events > Grade 2 occurred. The tumor control and low toxicity profile suggest BNCT could be a promising treatment modality in the management of early-stage cutaneous melanoma when a wide local excision is not feasible [40]. 

### 1.5. Brachytherapy (BT)

Skin brachytherapy (BT) (radionuclide or electronic) represents a standard-of-care option for appropriately selected patients with keratinocyte carcinoma (previously nonmelanoma skin cancer) [41]. There is no evidence in the literature regarding the experience of recurrent or metastatic melanoma treatment with BT. BT clinical application in malignant melanoma (MM) is supported by case reports and small mono-institutional studies, and its role remains undefined. [42,43,44]

Chadha et al. [42] used BT with temporary (iridium-192) and permanent (iodine-125) implants in the adjuvant setting after surgical excision of primary melanoma in the case of residual gross disease, microscopic residual disease, or clinically close margins of resection. Implants were applied at various body sites, such as in the extremities (14), intrathoracically (7), in the chest wall (2), intra-abdominally (2), gynecologically (6), and in the head and neck (2). Only patients with extremity lesions reported a high local control rate (93%), probably due to the site, which allowed for optimal surgical debulking and effective application of BT.

In a small experience of only four cases of primary melanoma with a Breslow index of at least 3 mm, due to the impossibility for the surgeon performing a small mutilating gesture or due to a contraindication to general anesthesia, interstitial BT with iridium-192 was chosen as a modality of RT [43]. The median total dose administered within the lesion was 64 Gy (range 60–70 Gy), and the margins were a median of 2.45 cm in the area (range of 2–3 cm) and 1.4 cm in depth (range of 1–2 cm). Excellent results in both the functional and the morphological plan were observed, without local recurrence at a median follow-up of four years. The choice of irradiation technique, BT rather than external radiotherapy in this series, depended on multiple criteria, such as the location of the lesion, its size and thickness, the patient’s age and distance from their home, possible co-morbidities, and the experience of the radiation oncologist. The authors concluded that the radiotherapy can be considered when surgical excision cannot be conservative and/or when the general condition of the patient is incompatible with general anesthesia. In all cases, the choice of treatment requires a confrontation with a multidisciplinary team.

High dose rate (HDR) surface-mold BT with iridium-192 was used to treat macroscopic regional cutaneous nodular metastases from melanoma in six patients [44]. Five out of six patients were treated with a dose of 30 Gy in five fractions (two frs/week) in a wide field and with an additional 6 Gy boost to macroscopic nodules. The dose was prescribed to a depth of 3–5 mm below the skin surface and the skin received approximately 35 Gy in five fractions at 7 Gy per fraction. Another patient received 36 Gy in six fractions followed by a 6 Gy boost to tumor nodules. There was a CR in all patients without in-field recurrence. The Radiation Therapy Oncology Group (RTOG) early and late toxicity was grade 1/2 in all patients except the one who received 36 Gy in 6 frs, who developed a small non-healing ulcer (grade 4 late skin reaction). This experience supports HDR surface mold BT as a valid treatment option for regional cutaneous nodular metastases located in the different skin surfaces of the body. The limitation of this technique is the lesion thickness, which must not be more than 7–10 mm, otherwise there is a risk of the lesion not being treating adequately and needing an increase in the dose to the skin, with greater side effects.

### 1.6. Radiotherapy and Immunotherapy

The introduction of immune checkpoint inhibitors (ICIs) against CTLA-4 (ipilimumab) and PD-1 (pembrolizumab and nivolumab) improved the prognosis of patients with advanced melanoma, demonstrating greater efficacy than chemotherapy [45].

However, patients may developed primary or acquired resistance to ICI, which may be partially overcome by tumor-targeted radiotherapy, which has multiple effects [46].

A large number of experimental data support the hypothesis that radiotherapy can transform the tumor into an in situ vaccine [47,48].

Radiotherapy promotes the cross-presentation of tumor cell antigens to T cells by inducing immunogenic cell death (ICD) [49]. ICD is defined by the production of molecular signals associated with cellular stress that can activate anti-tumor cytotoxic T cells capable of attacking the irradiated tumor as well as other metastatic sites outside the radiation field (abscopal effect phenomenon) [50,51].

Preclinical and clinical experiences have demonstrated the effectiveness of the combination of radiotherapy and immunotherapy, even if further information is necessary on the molecular and cellular circuits on the basis of which radiotherapy favors the elicitation of the specific tumor immune responses to improve the clinical effectiveness of the ICIs [51,52,53].

Many are still open issues, such as the correct radiation dose size and fractionation, the different response based on biological heterogeneity between the different tissues irradiated, the different tumor antigen expression, and the degree of immunosuppression present in the tumor microenvironment [49,54].

The field of immuno-radiotherapy is constantly evolving, and it is hoped that in the future it can also give results in the treatment of cutaneous and subcutaneous lesions and ITM.

### 1.7. Radiotherapy and Targeted Therapy

BRAF mutations are present in 40–60% of primary and metastatic melanomas (of which on average 80% carry the BRAFV600E variant) and are important for cell proliferation and resistance to apoptosis [7].

BRAF inhibitors (i) and MEKi are the first-line treatment of BRAF V600-mutant locally advanced/metastatic melanoma patients [45]

In vitro and in vivo experiences regarding the combination of BRAFi and RT suggest a radiosensitizing effect (cell-cycle arrest in G1, DNA repair decrease, restored radiation-induced apoptosis) that could improve tumor control but increase radiation-induced toxicity of healthy tissue [20,21,55,56,57].

Case reports and clinical series have documented significant skin toxicities (severe dermatitis, radionecrosis, and follicular cystic proliferation) that occurred, in the vast majority of cases, only in the irradiated areas of patients treated with a combination of RT and BRAFi, more so with vemurafenib than with dabrafenib [56,57].

Currently there is no clear indication of a correlation of the incidence or grade of radiation-related adverse effects and the dose of BRAFi, nor the correct timing of their combination. The common procedure to avoid or minimize radiation toxicities is intermittent dosing of BRAFi before and after RT (BRAFi 3 days before and after fractionated RT and 1 day before and after SRT/SRS) [55,58]

To date, however, there is no evidence that BRAFi transient interruption, discontinuation, or reduction in dosage might avoid the appearance of toxicities. On the contrary, an interruption or reduction of BRAFi dose during radiotherapy can elevate the risk of tumor progression [57].

Modern radiotherapy techniques such as stereotactic radiotherapy, with high ablative radiation dose and low toxicity profile, has increasingly been used in the treatment of melanoma metastases in association with BRAFi or BRAFi/MEKi [21].

Although the experiences in the literature of combining BRAFi and stereotactic radiotherapy in the treatment of intracranial melanoma metastases have increased in recent years, there are still few data on the treatment of extracranial metastases [21].

## 2. Conclusions

Cutaneous and subcutaneous melanoma lesions are a heterogeneous category due to different clinical presentation, location, symptoms, and prognosis. In this setting, radiotherapy plays a role when other local first-line treatments have failed or are not indicated and, although treatment protocols are not well defined, it is used to provide symptom relief and to prolong local tumor control. The choice of radiation dose, fractionation, and technique is based on the site, volume, and extent of the lesions, the patient’s performance status, the disease stage, and the technological availabilities.

The studies reported in this review demonstrate a close relationship between radiation dose fraction size, lesion volume, and local tumor response. Although there is no ideal fractionation, moderately high-dose and hypofractionated regimens appear to be more effective, especially in the treatment of limited-volume lesions. 

Numerous clinical experiences have shown that the addition of hyperthermia to radiotherapy, increasing local control without increasing toxicity, could be an effective treatment of cutaneous and subcutaneous melanoma lesions. However, these studies are old and heterogeneous in terms of techniques, doses, patient selection, site of treated lesions, and toxicity assessment, so it would be desirable to have prospective randomized studies, with modern and standardized techniques. In addition, the combination of RT and intralesional therapies seems to give encouraging results in terms of local control, but experiences in this field are still limited. 

BNCT appears to be a promising treatment modality in the management of cutaneous melanoma, but the number of patients treated with this method is still limited.

There is now ample evidence that demonstrate how radiotherapy in combination with immunotherapy can increase the tumor immune response and enhance the effectiveness of ICIs. For this reason, radiotherapy is increasingly acquiring a role not only in local control, but also in the systemic control of the disease combined with immunotherapy. 

The combination of radiotherapy with BRAFi and MEKi could improve tumor control but increase radiation-induced toxicity of healthy tissue, especially in the skin. 

Questions remain about the correct timing between the radiotherapy and immune-target therapy, the dose size, the fractionation, and the potential side effects of the combination.

More studies are needed to identify optimal radiotherapy use in subcutaneous melanoma therapy, either alone or in combination with other local and systemic therapies, in order to relieve symptoms and also improve local control and survival. 

In the treatment of cutaneous and subcutaneous melanoma lesions, since there are many local therapies alternative to radiotherapy and patients are often undergoing systemic therapy, treatment should be individualized after discussion with a multidisciplinary team of experts.

In the future, thanks to predictive models of treatment response and toxicity based on artificial intelligence algorithms (radiomics, genomics, proteomics, etc.) we will be increasingly able to personalize treatments and thus increase the survival and quality of life of our patients. 

## Figures and Tables

**Table 1:** Studies of radiotherapy in combination with hyperthermia in melanoma lesion treatment.

Author/Year	No. of Pts/ No. of Lesions Treated	Treatment	HT	RT (Total Dose/Dose Fr/No. of Frs/Days)	Results LC/OS	Factors Affecting Response Rate (Positively)	Toxicity of HT + RT
Overgaard (1995)	70/134	RT alone RT + HT	43 °C for 60 min within 30 min following RT	24 Gy/8 Gy/3/8 or 27 Gy/9 Gy/3/8	*RT alone* CR: 35% LC-2 y: 28% *HT + RT* CR: 62% LC-2 y: 46% 5-year OS 19% for all pts 38% for pts for whom all known diseases were controlled	Total radiation dose (≥27 Gy) and tumor volume (<4 cm).	No increase in acute/late radiation reactions Pain (6%)
Gonzalez Gonzalez (1986)	24/49	RT alone (8 l) RT + HT (38 l) HT alone (3 l)	43 °C within 30 min following RT	24 Gy/8 Gy/3/21 24 Gy/8 Gy/3/14 18 Gy/6 Gy/3/21 18 Gy/6 Gy/3/14 24 Gy/4 Gy/6/21 25 Gy/5 Gy/6/21 1 fr on day 1, 8, 14 or 1, 8, 21 or 2 frs per week in 21 days	*HT + RT* CR 50% in all pts CR 83% in pts treated with RT 8 Gy per fr *RT alone* CR 38% *HT alone* No response	Size of dose per fr (large) and tumor volume (small sizes).	Enhancement of acute skin reaction but not significant
Kim (1982)	38/100	RT alone RT + HT	30 min at 42.0–43.5 °C immediately prior to RT	42.90 Gy/3.3 Gy/13/twice per week or 40 Gy/4 Gy/10/ twice per week or 38.5 Gy/5.5 Gy/7/once per week or 39.6 Gy/6.6 Gy/6/once per week	Overall CR rate 75% HT + RT 46% RT alone (*p* < 0.01)	Frs per week (once per week regimen) and tumor volume (more than 10 cm^3^)	Not enhanced, normal tissue morbidity
Engin (1993)	126 (33 with MM)	RT alone RT + HT	45 °C (skin <43 °C) for 60 min within 15–30 min after RT	Mean RT dose 45 Gy ± 1 Gy (range, 13–80 Gy)/median dose 40 Gy/dose per fr in the range of 1.6–4.8 Gy/16 frs (range, 5–54 fractions)/35 days (range, 14–94 days)	CR 49% for all lesions 70% for superficial lesions 18% for deep lesions (*p* < 0.001) CR 36% for superficial deposits of MM OS 16.1 ±1.2 mo for superficial lesions 8.7 ± 1.1 mo for deep lesions (*p* = 0.00l)	Size of dose per fr (3–4 Gy), tumor volume . (<3 cm), and depth of lesions (superficial)	None (42, 33%) Erythema (59, 47%) Thermal blistering (25, 20%) Ulceration (27)
Emami (1988)	18/49 (28 CM or SM)	RT alone RT + HT	43 °C for 60 min, twice per week (72 h apart), for the duration of RT	Range 20–60 Gy/range 4–8 Gy	*Rt alone* CR: 23.9% PR: 34.3% *RT + HT* CR: 59.2% PR: 12.2%	Tumor volume (<3 cm)	-
Perez (1991)	307 *	RT alone RT + HT	43 °C for 60 min	32 Gy/4 Gy/8 frs/over 4 weeks	*RT alone* CR: 30% Tumors <3 cm: CR: 40% B CR: 0% T&E CR:38% H&N *RT + HT* CR: 32% Tumors <3 cm: CR: 62% B CR: 67% T&E CR:50% H&N	Tumor volume (<3 cm)	Thermal blistering (30%) No increase in acute/late radiation reactions

* The number of patients with MM lesions was not specified. RT: radiotherapy; HT: hyperthermia; N: number; pts: patients; LC: local control; OS: overall survival; CR: complete response; PR: partial response; y: years; frs: fractions; fr: fraction; l: localizations; min: minutes; MM: malignant melanoma; CM: cutaneous metastases; SM: subcutaneous metastases; mo: months; B: breast; T&E: trunk and extremities; H&N: head and neck.

**Table 2:** Studies of radiotherapy in combination with intralesional melanoma lesion treatment.

Author/Year	No. of pts/ No. of Lesions Treated	Treatment	RT (Total Dose/Dose fr/No. of frs/Days)	Results LC/OS	Factors Affecting Response Rate (Positively)	Toxicity
Foote (2017)	15/98	IL PV-10 + RT	30 Gy/5 Gy/6/2 frs/week over 3 weeks	ORR 86.6% (CR 33.3%+ PR 53.3%) Clinical benefit 93.3% (CR 33.3% +PR 53.3% + SD 6.7%) MSS (from the time of treatment) 12 mo 77% 24 mo 54% 36-mo 40% Median MSS 30.6 mo from the time of PV-10 treatment Median MSS 41.7 mo from the date of the primary melanoma diagnosis MSM 46.7%	Volume of lesions (≤10 mm)	G1-G2: Injection site pain (80%) Injection site swelling (60%) Injection site blistering (20%) Injection site erythema (20%) Injection site ulceration/bleeding (6.7%) Peripheral lymphedema (26.7%) Cellulitis (6.7%) G3: Injection site pain (6.7%) G4-G5: 0%
Paul (2003)	20/-	IL IFN-β +RT	40–50 Gy/1.8 Gy/5 days week	CR 70% PR 30% OS Pts with CR 4–93 mo (average 18 mo) Pts with PR 4–15 mo (average 8 mo)	-	-
Plesnicar (1982)	19/-	IL BCG +RT	The total dose and fractionation scheme was planned for each case individually and based on the degree and intensity of inflammatory response at the BCG injection sites.	groups: 1. Numerous and small (5 pts) CR: 100% 2. Multiple and large (9 pts) CR: 100% 3. Single or few, large (5 pts) CR: 0%, PR: 20%, None: 80%	Number and volume of lesions	-

RT: radiotherapy; IL: intralesional; N: number; pts: patients; LC: local control; OS: overall survival; CR: complete response; PR: partial response; SD: stable disease; y: years; frs: fractions; fr: fraction; mo: months; PV-10: Rose Bengal 4,5,6,7-tetrachloro-2,4,5,7-tetraiodofluorescein disodium; BCG: Bacille Calmette–Guérin; IFN-β: beta-interferon; ORR: overall response rate; MSS: melanoma-specific survival; MSM: melanoma-specific mortality; G: grade.
